# Osteopontin as a Biomarker in Interstitial Lung Diseases

**DOI:** 10.3390/biomedicines12051108

**Published:** 2024-05-16

**Authors:** David Iturbe-Fernández, Verónica Pulito-Cueto, Víctor M. Mora-Cuesta, Sara Remuzgo-Martínez, Diego J. Ferrer-Pargada, Fernanda Genre, Pilar Alonso-Lecue, Raquel López-Mejías, Belén Atienza-Mateo, Miguel A. González-Gay, José M. Cifrián-Martínez

**Affiliations:** 1 Pneumology Department, Marqués de Valdecilla University Hospital, 39008 Santander, Spain; vmoracuesta@gmail.com (V.M.M.-C.); diegojose.ferrer@scsalud.es (D.J.F.-P.); 2 Valdecilla Research Institute (IDIVAL), 39008 Santander, Spain; vpulito@idival.org (V.P.-C.); sararmtz@gmail.com (S.R.-M.); fernandagenre@gmail.com (F.G.); palonso@idival.org (P.A.-L.); rlopezmejias78@gmail.com (R.L.-M.); belen.atienza@scsalud.es (B.A.-M.); 3 Division of Rheumatology, Marqués de Valdecilla University Hospital, 39008 Santander, Spain; 4 Medicine and Psychiatry Department, University of Cantabria, 39005 Santander, Spain; miguelaggay@hotmail.com; 5 Division of Rheumatology, IIS-Fundación Jiménez Díaz, 28040 Madrid, Spain

**Keywords:** interstitial lung disease, idiopathic pulmonary fibrosis, biomarkers, osteopontin

## Abstract

Osteopontin (OPN) is a glycoprotein involved in Th1 and Th17 differentiation, and inflammation and tissue remodeling. OPN is a biomarker of disease activity in patients with autoimmune inflammatory conditions. This study aimed to assess the diagnostic and prognostic value of OPN in interstitial lung diseases (ILDs). Between May 2016 and October 2019, 344 patients with ILD were recruited at the Hospital Universitario Marqués de Valdecilla (Spain) and were prospectively followed-up. This study involved the determination of OPN serum levels by ELISA and OPN RNA expression quantified using qPCR. Six genetic polymorphisms in OPN (rs28357094, rs2853749, rs2853750, rs11728697, rs7695531, and rs1126616) were genotyped using TaqMan assays. OPN serum levels were also assessed in 140 healthy controls. OPN serum levels (median [interquartile range]) were significantly higher in ILD patients than in controls (1.05 [0.75–1.51] ng/mL versus 0.81 [0.65–0.98] ng/mL in healthy controls; *p* < 0.01). OPN serum levels were inversely correlated with the forced vital capacity. OPN serum levels were also higher in ILD patients who died or underwent lung transplantation when compared with the remaining ILD patients (1.15 [0.80–1.72] ng/mL versus 0.99 [0.66–1.32] ng/mL; *p* = 0.05). Survival worsened in ILD patients with OPN > 1.03 ng/mL at 1, 3, and 5 years. No statistically significant differences in the genetic frequencies of *OPN* polymorphisms or the RNA expression were found among the different ILD groups. Elevated levels of OPN in the serum may be a useful indicator in identifying patients with ILD who are more likely to experience poor outcomes.

## 1. Introduction

Interstitial lung diseases (ILDs) comprise a heterogeneous group of chronic pulmonary disorders characterized by fibrosis and inflammation of the interstitial space. All ILDs share similar clinical, functional, radiologic, and histologic features. According to their etiology, ILD can be classified as idiopathic interstitial pneumonia (IIP) and secondary ILD. Among IIP, idiopathic pulmonary fibrosis (IPF) is the most common and representative disease, and its course includes progressive scarring and a morphological pattern of usual interstitial pneumonia [1]. Patients with secondary ILD have a known cause and may be related to conditions such as connective tissue diseases (CTDs), inhaled antigens causing hypersensitivity pneumonitis, and drug toxicity. Despite their common characteristics, these entities differ significantly with respect to prognosis and therapeutic strategies. Therefore, a thorough differential diagnosis is crucial and often challenging. An accurate diagnosis can be achieved through multidisciplinary discussion involving specialists such as pulmonologists, radiologists, pathologists, and rheumatologists [2,3].

The severity of ILDs is highly variable and unpredictable. To date, no diagnostic assessment has been shown to predict individual clinical behaviors and help clinicians identify patients who will experience a more significant decline in lung function [4]. In this context, interest in serum biomarkers for diagnosis and prognosis of ILD is growing [5]. Most of the various biomarkers that have been investigated are associated with alveolar epithelial dysfunction, fibroproliferation, and immune system regulation [6]. However, given that the design and results of the published studies are often heterogeneous, more research is needed to obtain a better understanding of biomarkers in ILDs.

Osteopontin (OPN) is a highly phosphorylated glycoprotein involved in Th1 and Th17 differentiation, inflammation and tissue remodeling, biomineralization, cell viability, and wound healing [7]. OPN has both secreted and intracellular roles [8], and its involvement in pathophysiology has been studied in different conditions, including cancer, CTDs, cardiac fibrosis, diabetes, and cardiovascular diseases [7]. OPN is a chemotactic agent that recruits and activates various immune cells, such as lymphocytes, macrophages, neutrophils, and dendritic cells. It also regulates type 1 immune response through differential regulation of macrophage interleukin (IL) 12 and IL-10 cytokine expression [9].

*OPN* expression is associated with fibrosis, acting as a chemotactic factor for fibroblasts, modulating the secretion of metalloproteinases (MMPs), and regulating the production of the transforming growth factor (TGF)β [10,11]. However, little is known about the role of OPN in ILDs. In bronchoalveolar lavage (BAL) cells from patients with smoking-related ILDs, OPN production is increased, promoting the accumulation of macrophages and Langerhans cells [12]. Along with surfactant protein-D (SP-D) and MMP-7, it can enhance the accuracy of the differential diagnosis in patients with IPF, compared with non-IPF ILDs [13]. Additionally, *OPN* is the most up-regulated gene in the lungs of patients with IPF (levels up to 20-fold higher than in healthy lungs), and OPN levels in BAL fluid are higher in IPF patients than in healthy controls [11]. In patients with anti-MDA5 antibody-positive dermatomyositis-associated with ILD, OPN is overexpressed in epithelial cells and macrophages; therefore, it could be used as a prognostic marker [14].

According to a systematic literature review and meta-analysis, studies of OPN as a biomarker of IPF were based on small samples and reached inconsistent conclusions [15]. An ideal biomarker for IPF should be dysregulated in disease but not in a healthy state. In addition, it should be phenotype specific, able to predict response to treatment, and non-invasively obtained [16].

In this study, we aimed to assess the diagnostic and prognostic value of serum OPN levels, OPN RNA expression, and OPN gene polymorphisms in a diverse population of ILD patients.

## 2. Methods

### 2.1. Study Population

We performed a prospective, observational study. The study population comprised 344 patients from the ILD unit at University Hospital Marqués de Valdecilla (Santander, Spain) who were included between May 2016 and October 2019. All patients fulfilled the American Thoracic Society/European Respiratory Society classification and diagnostic criteria for ILD [1,3]. No other inclusion or exclusion criteria were defined. Clinical, demographic, and functional data were obtained from medical records and a specifically designed database. All the diagnoses were confirmed after a multidisciplinary discussion, according to the classification used at the time [17]. To compare with ILD, we also assessed 140 healthy controls matched by age and sex.

The inclusion criteria were a diagnosis of ILD, ≥18 years of age, and a follow-up time of six months as a minimum. The exclusion criteria were ≤18 years of age or any active systemic disease such as malignancy, acute infections, and liver or kidney disease. The inclusion criteria for healthy volunteers were the absence of chronic illnesses and abnormalities in pulmonary function tests. This study was approved by the local institutional ethics committee and was conducted in line with the ethical standards of the approved guidelines and regulations and according to the Declaration of Helsinki. All enrolled subjects agreed to participate and signed the informed consent form.

### 2.2. Pulmonary Function Testing

Pulmonary function tests (PFTs) included forced vital capacity (FVC), forced expiratory volume in 1 s (FEV_1_), and diffusing capacity of the lung for carbon monoxide (DLCO). PFTs were performed at baseline (the time when the blood sample was taken) and every 3 to 6 months, depending on the clinical indication. Medical records were also reviewed to obtain information on PFT values before recruitment. Lung function variation was assessed based on changes in FVC 12 months before and after baseline. A decrease in more than 10% in FVC, either in the 12 months before or after baseline, was expressed as a dichotomous variable.

### 2.3. Genotyping of OPN Polymorphisms

Genomic DNA was extracted from peripheral blood using the REALPURE SSS kit (RBME04, REAL, Durviz S.L., Valencia, Spain). The quality and quantity of extracted DNA were measured in a spectrophotometer (NanoDrop^®^ ND-1000, Nanodrop Technologies, Wilmington, DE, USA). Furthermore, 6 polymorphisms in *OPN* (rs28357094, rs2853749, rs2853750, rs11728697, rs7695531, and rs1126616) were genotyped using TaqMan^TM^ SNP Genotyping Assays (Thermo Fisher Scientific Inc., Waltham, MA, USA). Negative controls and duplicate samples were included to check the accuracy of genotyping. All genotype data were checked for deviation from the Hardy–Weinberg equilibrium.

### 2.4. OPN mRNA Expression

Total RNA was isolated from peripheral blood using the NucleoSpin RNA Blood Kit (Macherey-Nagel GmbH & Co. KG, Düren, Germany) according to the manufacturer’s instructions. RNA was reverse transcribed into complementary DNA (cDNA) using the iScript™ Advanced cDNA Synthesis Kit for reverse transcription-qPCR (Bio-Rad, Hercules, CA, USA). Then, cDNA was amplified using qPCR in the thermocycler QuantStudio^TM^ 7 Flex Real-Time PCR System with SsoAdvanced^TM^ Universal SYBR^®^ Green Supermix (Bio-Rad, Hercules, CA, USA). All samples were assayed in duplicate, and experimental control assays were included. Relative *OPN* mRNA expression was analyzed by the comparative Ct method using *GAPDH* as the housekeeping gene.

### 2.5. Serum OPN Levels

Serum OPN levels were determined using a commercial enzyme-linked immunosorbent assay (ELISA) kit (ab100618, Abcam, Cambridge, UK) in accordance with the manufacturer’s instructions. All the samples were analyzed in duplicate and quantified relative to a standard curve using 4-parameter logistic regression with MyAssays^®^ online (http://myassays.com/, accessed on 1 May 2023) software, as recommended by the manufacturer.

### 2.6. Statistical Analysis

The statistical analysis was conducted using IBM SPSS Statistics for Windows, Version 20.0 (IBM Corp., Armonk, NY, USA). Categorical variables were reported as frequency (n) and percentage (%). Continuous variables were expressed as mean ± standard deviation (SD) or median and interquartile range (25th and 75th) depending on whether their distribution was normal or not according to the Shapiro–Wilk test. The chi-square test was performed to explore the association between 2 qualitative variables. The *t* test for independent samples or the Mann–Whitney test was used to analyze differences between quantitative and dichotomous variables. The Kruskal–Wallis test was used to assess differences between continuous variables across several groups. The correlation between continuous variables was determined using Pearson’s r or Spearman’s rho (S_r_), depending on whether the distribution was normal or not. Receiver operating characteristic (ROC) curve analysis was performed to estimate an optimal threshold and the diagnostic performance of serum OPN levels for identifying patients with worse survival. Kaplan–Meier graphs were constructed to represent cumulative survival, and a logrank test was carried out to analyze differences between groups. For allelic and genotypic analysis, a chi-squared test was used to compare between groups. The strength of association was estimated using odds ratio (OR) and 95% confidence intervals (CI). Additionally, allelic combination (haplotype) analysis for the OPN polymorphisms evaluated was carried out. Haplotype frequencies were calculated using the Haploview v4.2 software (http://broad.mit.edu/mpg/haploview accessed on 18 May 2024) and a chi-squared test was used to compare between groups. The strength of association was estimated by OR and 95% CI. A *p* value ≤ 0.05 was considered statistically significant in all cases.

## 3. Results

### 3.1. Characteristics of the Study Population

We included 344 patients with ILD. The median age of the patients at the time of this study was 62 (56–67) years. Almost 70% were men, and 66.7% had a history of smoking. Definitive diagnoses were made after multidisciplinary discussion, although the diagnosis of ILD was exclusively clinical–radiological in 40% of patients. The most common diagnoses were IPF and ILD related to a CTD (Table 1). It is noteworthy that 95 patients (27.6%) were recruited after lung transplantation and were evaluated for genetic polymorphisms only. Therefore, variations in PFT results during the year before and after baseline were only assessed in patients who did not undergo lung transplantation (Table 2).

### 3.2. Polymorphisms in OPN

Six genetic polymorphisms in *OPN* (rs28357094, rs2853749, rs2853750, rs11728697, rs7695531, and rs1126616) were assessed. In this sense, we analyzed the potential differences in the OPN polymorphism, genotype, and allele frequencies between patients with ILD, stratified according to the subtypes of ILD. No statistically significant differences were disclosed. Additionally, the haplotype analyses did not yield additional information since haplotype frequencies of OPN did not differ between patients with different subtypes of ILD.

### 3.3. OPN RNA Expression

Median OPN mRNA expression was 0.00147 (8.27 × 10^−4^–2.76 × 10^−3^) in ILD patients. No differences in OPN mRNA expression were observed in different types of ILDs. Furthermore, no correlation was found between OPN serum levels and OPN mRNA expression in ILD patients.

### 3.4. Serum OPN Level

Median OPN levels were significantly higher in ILD patients than in healthy controls [1.05 (0.75–1.51) ng/mL versus 0.81 (0.65–0.98) pg/mL, respectively] (*p* < 0.01).

We did not observe any differences in serum levels of OPN when the ILD cohort was categorized based on different subtypes of ILD.

### 3.5. Serum OPN Level and Pulmonary Function Tests

At baseline, the serum OPN level was inversely correlated, albeit weakly, with FVC% (S_r_ = −0.13; *p* = 0.028) but not with DLCO% (*p* = 0.34).

An inverse correlation was found between OPN levels and variation in FVC during the year before baseline (S_r_ = −0.23; *p* < 0.01), but not during the year after (*p* = 0.7).

The median OPN level was statistically significantly higher 1 year before and after baseline in patients with FVC decrease > 10% compared to <10% (Table 3).

### 3.6. Serum OPN Level and Time to Death or Lung Transplantation

The median survival (time to death or lung transplantation) for ILD patients overall was 8.25 (3.89–16.61) years. The median OPN level was 1.15 (0.80–1.72) ng/mL in patients who died or underwent lung transplantation and 0.99 (0.66–1.32) ng/mL in survivors (*p* = 0.05).

**Figure 1:**
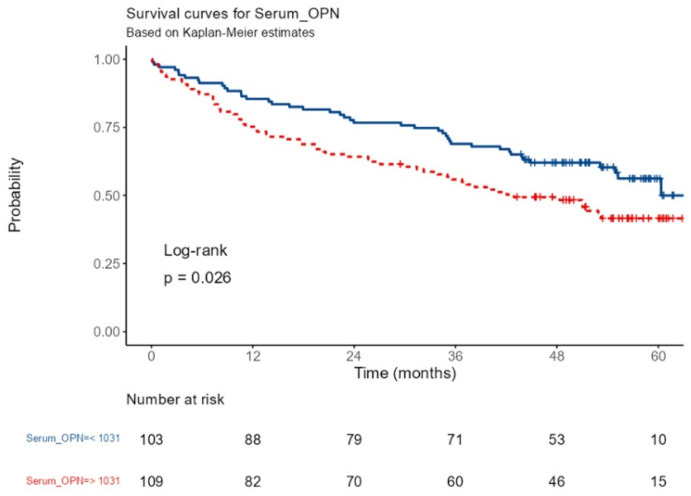
Survival (time to death or lung transplantation) according to the serum OPN level in 212 patients with ILD.

After performing an ROC curve analysis, we estimated that the cutoff point with the highest sensitivity and specificity was 1.03 ng/mL. Using this threshold to obtain a dichotomous variable, we found statistically significant differences in 1-, 3-, and 5-year survival. The 1-, 3-, and 5-year survival for patients with OPN ≥ 1.03 ng/mL was 75.2% (67.6–83.8%), 55.9% (47.3–66%), and 41.6% (32.9–52.6%), while it was 85.4% (78.9–92.5%), 68.9% (60.6–78.5%), and 56.23% (46.6–67.9%) for patients with OPN < 1.03 ng/mL (Figure 1).

## 4. Discussion

Our study aimed to assess the value of OPN as a biomarker in ILDs. The serum OPN level was higher in patients with ILDs than in healthy controls. While OPN can prove helpful in the diagnosis of ILDs, it should be considered together with other investigations because OPN can be increased in various conditions. No correlation was found between serum OPN levels and RNA expression. Moreover, no differences were found in serum OPN levels or RNA expression between the different diagnoses of ILD. Hence, we concluded that these parameters are not beneficial in distinguishing among different ILDs. However, the serum OPN level was related to pulmonary function. We recorded a weak but statistically significant negative correlation between serum OPN levels and FVC% at baseline. There was also a weak but statistically significant negative correlation between serum OPN levels and FVC loss 1-year before baseline. However, this correlation was not found for FVC during the year after baseline.

The potential usefulness of OPN as a prognostic biomarker is suggested by the change in serum OPN levels in patients who lost more than 10% of FVC both one year before and one year after baseline. In these patients, the serum OPN level was higher than in patients who experienced minor losses, with statistically significant differences.

Another finding on the predictive value of OPN was that a serum level > 1.03 ng/mL predicted survival (time to transplantation or death) at 12, 36, and 60 months. A previous study showed that the mortality risk increased in 71 patients with IPF if the serum OPN level was >3.24 ng/mL (*p* = 0.019) [18]. Although this value was higher than in our study, there were some methodological differences as the endpoint was mortality in patients with stable or exacerbated IPF, whereas in our study, we assessed the risk of transplantation and mortality in patients at baseline.

We did not find any difference in *OPN* RNA expression between the different types of ILD. Furthermore, no correlation was found between serum OPN levels and RNA expression. We did not investigate a correlation between *OPN* RNA expression and prognosis in patients with ILD. However, in the above-cited systematic review and meta-analysis, using data from the Gene Expression Omnibus (GEO) database, *OPN* expression was higher in patients with IPF than in healthy controls or patients with lung cancer. Furthermore, higher *OPN* expression predicted a poorer prognosis in patients with IPF (HR = 1.42; 95% CI = 1.27, 1.58; *p* < 0.01) [15].

The role of OPN has been described in diverse fibrotic conditions. In systemic sclerosis (SSc)-related ILD, the secretion of OPN by macrophages has a significant impact on the process of tissue remodeling, as it sensitizes and mobilizes fibroblasts in response to other growth factors that promote fibrosis. Consequently, this intricate mechanism contributes to the progression of fibrosis [19]. *OPN* expression was found to be increased in SSc skin in a research investigation that employed microarray analyses to compare SSc skin with healthy control skin [20]. Additionally, OPN-deficient mice exhibited a reduction in the thickness of the skin and the development of fibrosis in the dermis, as compared to wild-type mice treated with bleomycin. This phenomenon may potentially be attributed to the alteration in the production of TGF-β [21].

OPN is also related to liver fibrosis. Urtasun et al. demonstrated that mice genetically modified to overexpress the *OPN* gene exhibited a spontaneous occurrence of liver fibrosis. In comparison, hepatic stellate cells (HSCs) obtained from wild-type mice demonstrated a more pronounced phenotype associated with the development of fibrosis compared to those isolated from mice lacking the *OPN* gene. This phenomenon was facilitated through the activation of the phosphoinositide 3-kinase (PI3K)/phosphorylated protein kinase B (pAkt)/nuclear factor kappa B (NF-κB) signaling pathway, which was initiated by the interaction between OPN and αvβ3 integrin on the membrane of the HSCs. This interaction led to the increased expression of collagen type I (COL-I), which subsequently contributed to the progression of [22].

The development of kidney tubulointerstitial fibrosis is induced by OPN, which enhances the infiltration of macrophages. Research has demonstrated that mice lacking OPN and undergoing unilateral ureteral obstruction display diminished macrophage influx, collagen deposition, and TGF-β1 mRNA expression in comparison to wild-type mice [23,24]. OPN’s N-terminal fragment (N-OPN) was up-regulated in various animal models of renal diseases and could be contained by tubular exosomes. Consequently, OPN and N-OPN were transported to fibroblasts in tubular cell-derived exosomes, where they interacted with CD44 and enhanced fibroblast proliferation and activation [25].

On the heart, OPN is associated with the activation of cardiac myofibroblasts. Its profibrotic effect may be linked to its prothrombin-mediated cleavage. In an animal heart model, OPN was increased after pressure overload. However, OPN only induced COL-I expression after it was cleaved by thrombin [26].

Our study was limited by the absence of inclusion and exclusion criteria, which could lead to selection bias. In addition, we did not compare OPN levels in patients exposed and not exposed to antifibrotic drugs, because OPN levels have been shown to predict different transplant-free survival in patients with IPF according to whether they were treated or not with antifibrotic drugs [27]. Additionally, the analysis did not take tobacco use into account, which can be an important factor in the pathophysiology of ILD. Therefore, tobacco cessation should always be recommended for these patients [28,29].

In patients with connective tissue diseases and acute-onset diffuse ILD, the serum OPN level was significantly increased in those who later died than in those who survived. This difference was also seen for serum levels of tissue inhibitor of metalloproteinase (TIMP)-1, MMP-9, interleukin2 receptor α (IL-2Rα), IL-1 receptor antagonist, MMP-1, and MMP-8 [30].

Given that OPN may be increased in other conditions, it can be combined with other biomarkers to enhance its predictive value in ILDs [7]. White et al. [13] measured levels of OPN, MMP-7, and SP-D in patients with IPF (n = 149), rheumatoid arthritis-associated ILD (n = 33), alternative idiopathic ILDs (n = 41), and healthy controls (n = 127). Their results suggested that an index comprising OPN, MMP-7, and SP-D could differentiate IPF from other ILDs and healthy controls [13]. In a study of 516 IPF patients from three different cohorts, a progression index comprising OPN, MMP-7, intercellular adhesion molecule-1 (ICAM-1), and periostin predicted the risk of progression, mortality, and progression-free survival in patients with IPF [31].

## 5. Conclusions

In conclusion, this study provided data on OPN as a biomarker in ILDs that could be useful when designing new diagnostic strategies and prognostic tools, either when used alone or when combined with other biomarkers.

## Figures and Tables

**Table 1:** Demographics and clinical characteristics (n = 344).

Variable	Value, n (%), Median (IQR) or Mean ± SD
Age, years	62 (56–67)
Sex, male	232 (67.4)
Smoking habit	
Non-smokers	115 (33.3)
Ex-smokers	227 (66.1)
Current smokers	2 (0.6)
Diagnostic method, n (%)	
Clinical and radiological	139 (40.4)
Histological analysis	
Surgical biopsy	111 (32.3)
Transbronchial biopsy	52 (15.1)
Cryobiopsy	24 (7.0)
Explant (after lung transplantation)	18 (5.2)
ILD diagnoses, n (%)	
IPF	123 (35.8)
CTD-ILD	71 (20.6)
CHN	38 (11.0)
Unclassifiable	37 (10.8)
NSIP	21 (6.1)
LCH	14 (4.1)
LAM	8 (2.3)
Sarcoidosis	7 (2.0)
SR-ILD	5 (1.5)
Pneumoconiosis	4 (1.2)
PPFE	4 (1.2)
Asbestosis	3 (0.9)
HPS	3 (0.9)
DR-ILD	2 (0.6)
LIN	1 (0.3)
COP	1 (0.3)
RT-ILD	1 (0.3)
LN	1 (0.3)
Pulmonary function tests at baseline *	
FVC, %	74.8 ± 23
FEV_1_, %	73 ± 21.9
FEV_1_/FVC, %	78.7 ± 10.2
DLCO, %	30.9 (21.4–42.9)
Time to death or lung transplantation, * years	8.25 (3.9–16.6)

* **The 95 patients who underwent lung transplantation were not included in the analysis.** ILD: interstitial lung disease, IPF: idiopathic pulmonary fibrosis, CTD-ILD: connective tissue disease-ILD, CHN: chronic hypersensitivity pneumonitis, NSIP: non-specific idiopathic pneumonia, LCH: Langerhans cells histiocytosis, SR-ILD: smoking-related ILD, PPFE: pleuroparenchymal fibroelastosis, HPS: Hermansky-Pudlak syndrome, DR-ILD: drug-related ILD, COP: cryptogenic organizing pneumonia, RT-ILD: radiotherapy-related ILD, LIN: lymphoid interstitial pneumonia, LN: lipoid pneumonia, FVC: forced vital capacity, FEV_1_: forced expiratory volume in 1 s, and DLCO: diffusing capacity of the lung for carbon monoxide.

**Table 2:** Variations in pulmonary function tests, before and after the biomarker was evaluated (baseline).

	n	Median Change in FVC	Decrease > 10% in FVC
**1 year before baseline**	137	−5.28 mL (−16.3–10.9)	23 patients (18.9%)
**1 year after baseline**	187	−3.84 mL (−16.4–11)	29 patients (18.1%)

FVC: forced vital capacity.

**Table 3:** OPN levels 1-year before and after baseline according to decrease in FVC.

		OPN Level (ng/mL), Median (Percentiles)			
	n	>10% Decrease in FVC	n	≤10% Decrease in FVC	*p*
**1 year before baseline**	23	1.22 (1.01–1.47)	99	1.00 (0.75–1.30)	0.029
**1 year after baseline**	29	1.23 (1.34–1.77)	131	0.99 (0.73–1.40)	0.038

FVC: forced vital capacity.

## Data Availability

The data that support the findings of this study are available from the corresponding author upon reasonable request.
